# Rare osteosarcoma cell subpopulation protein array and profiling using imaging mass cytometry and bioinformatics analysis

**DOI:** 10.1186/s12885-020-07203-7

**Published:** 2020-07-31

**Authors:** Izhar S. Batth, Qing Meng, Qi Wang, Keila E. Torres, Jared Burks, Jing Wang, Richard Gorlick, Shulin Li

**Affiliations:** 1 Department of Pediatrics-Research, Division of Pediatrics, The University of Texas MD Anderson Cancer Center, 1515 Holcombe Blvd, Houston, TX 77030 USA; 2 Department of Laboratory Medicine, Division of Pathology and Laboratory Medicine, Houston, USA; 3 Department of Bioinformatics and Computational Biology, Division of Science, Houston, USA; 4 Department of Surgical Oncology, Division of Surgery, Houston, USA; 5 Department of Leukemia, Division of Cancer Medicine, UT MD Anderson Cancer Center, 1515 Holcombe Blvd, Houston, TX 77030 USA

**Keywords:** Imaging mass cytometry (IMC), Circulating tumor cells (CTCs), T-distributed stochastic neighbor embedding (t-SNE), Patient-derived xenograft (PDX), Copy number variations (CNV), Fluorescence associated cell-sorting (FACS), Fine needle aspirates (FNA), Cytometry time-of-flight (CyTOF), Cell surface vimentin (CSV), Smooth muscle actin (SMA)

## Abstract

**Background:**

Single rare cell characterization represents a new scientific front in personalized therapy. Imaging mass cytometry (IMC) may be able to address all these questions by combining the power of MS-CyTOF and microscopy.

**Methods:**

We have investigated this IMC method using < 100 to up to 1000 cells from human sarcoma tumor cell lines by incorporating bioinformatics-based t-Distributed Stochastic Neighbor Embedding (t-SNE) analysis of highly multiplexed IMC imaging data. We tested this process on osteosarcoma cell lines TC71, OHS as well as osteosarcoma patient-derived xenograft (PDX) cell lines M31, M36, and M60. We also validated our analysis using sarcoma patient-derived CTCs.

**Results:**

We successfully identified heterogeneity within individual tumor cell lines, the same PDX cells, and the CTCs from the same patient by detecting multiple protein targets and protein localization. Overall, these data reveal that our t-SNE-based approach can not only identify rare cells within the same cell line or cell population, but also discriminate amongst varied groups to detect similarities and differences.

**Conclusions:**

This method helps us make greater inroads towards generating patient-specific CTC fingerprinting that could provide an accurate tumor status from a minimally-invasive liquid biopsy.

## Background

Circulating tumor cells (CTCs) are rare cells that have been repeatedly demonstrated to contain predictive properties for patient survival [1–3]. The allure of CTCs is their key role as representatives of the source tumors. Capture and analysis of these rare cells by way of liquid biopsies can help scientists and clinicians obtain a snapshot of the tumor’s status [4]. Indeed, repeated studies with large cohorts of multiple tumor types have consistently shown higher CTC enumeration to be associated with worse patient progression-free and overall survival [5–8]. The relatively easy methods of collecting these cells allow for fast processing and information acquisition. While the capture and imaging of CTCs reveals valuable information regarding surface markers and abundance, the amount of data that can be collected by these methods per cell is highly limited. A key requirement for accurate and reliable analysis of CTCs is the ability to discern and identify unique cells from extremely small sample sizes because the number of CTCs isolated out of a single vial of blood (up to 10 ml) is about a few to only a few 100 at the most. Therefore, how to effectively use the few CTCs to obtain maximum tumor cell information becomes a high interest of research. Highly sensitive methods such as single cell RNA sequencing and exome sequencing can provide transcriptional information [2, 9]. Correlating known genetic aberrations such as copy number variations (CNVs) associated with tumor prognosis and physiological states allows for accurate and reliable assessment of patient outlook [10, 11]. However these techniques are highly cost- and labor-intensive. Further, the isolation of rare cells into separate chamber adds additional steps requiring specialized equipment such as the fluorescence associated cell sorting (FACS), or DepArray [2]. Of note, this approach cannot account for functionally relevant levels of proteins unless one decides to follow through with a complicated single cell western blot [12]. In some cases, CTC expansion may be needed but CTCs expansion seems only works in a few tumor types based on the reports [13–15]. Even if these assays are successful against all CTCs from any tumors, the protein information (quantity, modification, and localization) cannot be addressed by these methods. Microscopy methods can address these questions but only a few proteins can be analyzed for each single CTC cell.

Fine needle aspirates (FNAs) are a commonly used method to extract rare tissue for tumor assessment [16, 17]. This invasive procedure is necessary to accurately determine tumor grade and relevant information such as gene expression and genetic changes in tumor cells [17]. Compared to CTCs, the cell number is less a limiting factor but the same limiting factor for detecting protein localization and large number of proteins in each cell still exist.

To obtain a several folds higher multiplexed labeling with a similar approach we turned towards the recently developed Imaging mass cytometry (IMC) technology [18]. Cytometry time of flight (CyTOF) is a highly advanced flow cytometry-based (called mass cytometry) technology that can process cells appended with far greater number of antibodies as conventional flow cytometry [19]. The multiplex labeling is enabled by using metal ions, rather than fluorescent molecules as reporting markers on antibodies. As with any other flow-based method, this protocol requires a large number of cells (> 10,000) for proper analysis which is not feasible for rare cell analysis such as CTCs [20]. IMC allows for several-fold greater multiplexed labeling compared to conventional immunofluorescence techniques. This approach allows for the theoretical possibility of simultaneous detection of over 135 various markers in a single cell per experiment [18, 20–22]. This can provide significantly greater protein quantification and co-localization information compared to conventional methods. However, the research on using IMC for rare cell study is barely seen except for one [23]. Our goal in this report is not only to utilize this IMC imaging method but also integrating bioinformatic analytical tools for rare cell characterization and profiling.

To accomplish our goal, we first targeted our approach using human tumor cell lines with a low number (< 100) to assimilate rare cell case and determine feasibility. We also used rare CTCs from patient blood samples as real case study. In the low cell number analysis, we discovered that the colocalization of cell surface vimentin (CSV) positive cells with non CSV expressing cells created a unique protein signature via bioinformatics analysis such as t-Distributed Stochastic Neighbor Embedding (t-SNE) clustering, correlation matrix, paired scatter plot, etc. [24]. These methods were incorporated into IMC image data interpretation in this study. Similar finding in PDX cell lines at low number reinforced the validity of our approach which was later applied to human CTCs. Interestingly, we found that CSV+ CTCs from the same patient are relatively homogeneous, while CTC comparison across patients showed heterogeneity. CSV+ tumor cells show significant difference from CSV- cells in smooth muscle actin (SMA) expression.

## Methods

### Cell lines and patient samples

OHS (RRID:CVCL_B450) and TC71 (RRID:CVCL_2213) cell lines were obtained from NCI depository. Therapy-resistant PDX cell lines M31, M36, and M60 were generously provided by Dr. Richard Gorlick [25]. All cells were treated with mycoplasm removal agent for 2 weeks prior to use. Peripheral blood from patients with metastatic sarcoma was provided by Dr. Keila E. Torres. Informed consented patient sample collection for CTC analysis is approved under MD Anderson institutional review board protocol PA13–0014.

#### CTC isolation and labeling

We use an in-house developed CTC-targeting antibody, 84–1, which is specific for CSV. The procedure for isolating CTCs from whole blood has been previously described [4]. Briefly, whole blood is subjected to gradient centrifugation to isolate the peripheral blood mononuclear cells (PBMCs). These cells are processed to first remove the CD45 positive population then select the CSV positive cells using our 84–1 antibody. After isolation, we adopted the standard antibody staining methods for IMC-based metal-conjugated antibodies. The isolated cells are labeled with the metal-conjugated 84–1 antibody in solution and cytospun on to polylysine-coated slides using specially modified, narrow-funnel caskets. After fixation and blocking, cells are labeled with desired targets (maximum 37) on the slide. We refined our approach to stain 84–1 in solution through a trial and error process which minimizes non-specific labeling that had been previously observed on PBMC background. IMC-labeled cells on poly-lysine coated slides were imaged on Fluidigm Hyperion Imaging System (Fluidigm, South San Francisco, CA, USA).

### Analytical methods

The analyses were performed using R language. Euclidean distance was used in the dendrogram in the cluster heat map. Power analysis was performed using PWR package. t-Distributed Stochastic Neighbor Embedding (t-SNE) was performed using tSNE package with the default parameter setting. The same t-SNE analysis was performed 10 times to confirm the consistency despite the stochastic nature of the method.

#### IMC imaging and processing

Fluidigm Hyperion Imaging Mass Cytometer System at the UT MD Anderson flow cytometry & cell imaging core facility was employed for the laser-based cell ablation and imaging (1 μm resolution). Channel-specific signal data is gathered on a per-pixel basis. We used the BitPlane IMARIS software analysis package to mask pixel data into single cell data. These aggregations of pixels were then used to record the signal localization (corresponding to nuclear, cytoplasmic, and membrane) and intensity per individual cells. Cellular regions were identified by correlating with Ir191/193 which labels DNA, smooth muscle actin (SMA) which labels membrane, and using ImarisCell to mask and identify the region between the nucleus and membrane, which was labeled the cytoplasm. Data shown in heat map is normalized to nuclear labeling signal strength.

The antibodies used for this study are listed in Table 1.

**Table 1:** List of antibodies and corresponding metal isotope labels used in study

Target	Label	Source	Catalog#
PTEN	141Pr	BioLegend	655,002
Smooth muscle actin	141Pr		
Cell Surface Vimentin	142Nd	Li Lab	
p53	143Nd	DVS-Fluidigm	3143026D
PD-L1	150Nd	DVS-Fluidigm	3150031D
p21	154Sm	Sigma	P1484
Src	155Gd	CST	2109BF
p-P38 MAPK	160Gd	CST	4511BF
PDGFRβ	161Dy	BioLegend	323,602
mTOR	162Dy	GenScript	A01154
c-Myc	163Dy	CST	5605BF
p-Src(Tyr416)	164Dy	CST	6943BF
β-catenin, active (non-phospho)	165Ho	DVS-Fluidigm	3165032D
IL-10	166Er	DVS-Fluidigm	3166008B
TGF-β1	169Tm	BioLegend	349,702
Caspase 3 (cleaved)	172Yb	DVS-Fluidigm	3172027D
EpCAM/CD326	174Yb	BioLegend	324,202
TNFα	175Lu	DVS-Fluidigm	3175023B

## Results

### Method development

**Fig. 1:**
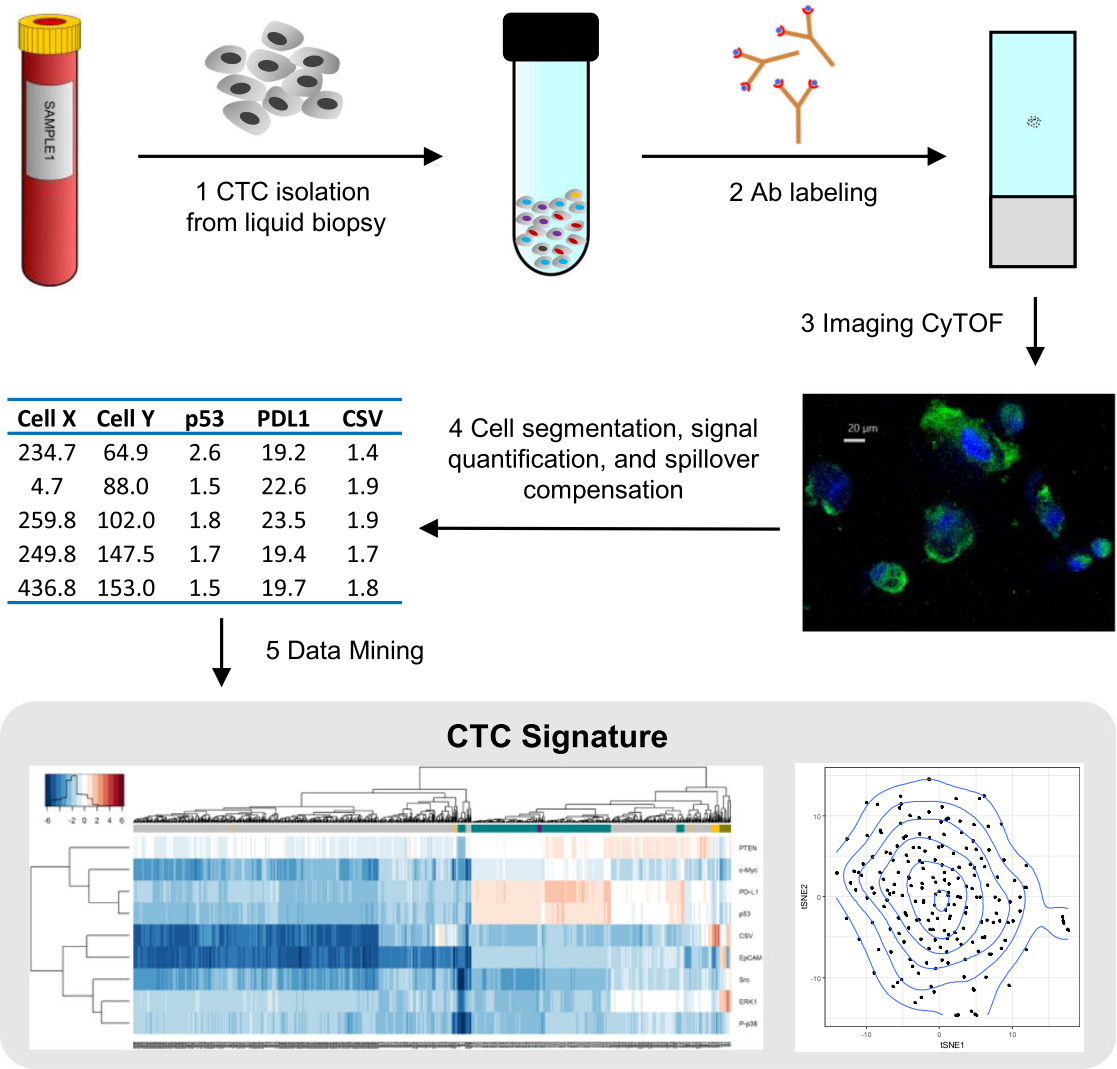
(1.5 column): Workflow for rare cell isolation and analysis. Liquid biopsy is processed for CTC isolation. Afterwards, CTC are cytospun on to glass slides and labeled with metal-conjugated Abs then imaged on the Fluidigm Hyperion Imaging system. The image is analyzed and the signal is quantified for bioinformatics-based analysis to detect and determine a unique patient-based CTC signature

**Fig. 2:**
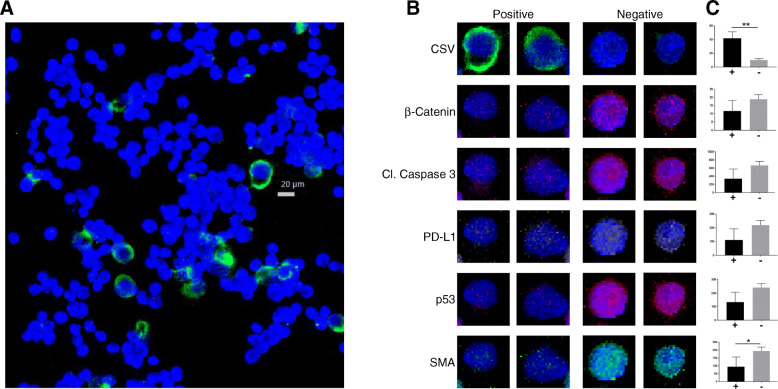
(2 columns): Rare cell identification. Unique staining properties of identified CSV+ cells in a cell-line panel illustrates the associated difference in other proteins compared to CSV- cells. **a** OHS cell line IMC labeled for nucleus (blue) and CSV (green). **b** High and low CSV cells individually studied for relationship between CSV and selected proteins. Far right shows plot of each cell’s quantified value of a given protein (x-axis) against CSV (y-axis); left bar is CSV+ right bar is CSV-. **p ≤ 0.006; *p ≤ 0.05

**Fig. 3:**
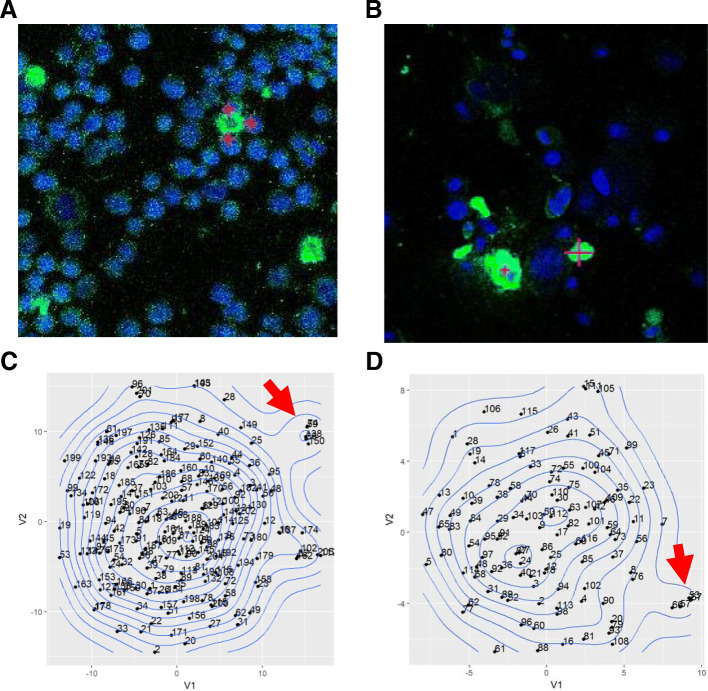
(2 columns): Cell lines imaged by IMC. Cells representing sarcoma cell line TC71(**a**), and osteosarcoma PDX cell line M36(**b**) were imaged. Identified outliers are marked by a magenta-colored cross. tSNE plot with density map highlighting outliers from the main population are shown for TC71 and M36 in panels (**c**) and (**d**), respectively; red arrows in panels (**c**) and (**d**) indicate outlier populations. Blue stain is nucleus; Green stain is CSV. Images are representative of multiple panels

To be able to more easily detect and discern protein signature in rare cells such as CTCs (out of less than 100 cells) than conventional techniques such as flow cytometry and confocal imaging, we first used sarcoma cell lines TC71 and OHS as platforms for method development. We chose to begin with cell lines since they are easily available and we’ve previously found that even in cell lines, there is inherent heterogeneity in CSV protein expression [26]. We asked whether this inherent heterogeneity (whether it is based on CSV or other protein) in tumor cells can be further elucidated with multiplexed antibody labeling. Conventional imaging methods rely on four to six channel filters to detect as many targets for study. Other means such as flow cytometry are not feasible for rare cell detection, as a large sample size is required (10,000 or more). Therefore, we employed the recently developed IMC approach which allows for highly multiplexed imaging. We modified standard cell cytospinning process to construct a narrow load inlet and a 10 mm outlet channel to focus the flow of cells on to the slide. Fewer than 500 TC71 and OHS sarcoma cells were labeled with metal-conjugated antibodies to test for the ability to study intercellular protein level variations in rare cell populations. The process for labeling cell line samples is similar to the workflow shown in Fig. 1 with the exception of replacing CTCs with cell line cells. To detect the tumorigenic potential of the cells, we chose antibodies representing several pathways related to tumor propagation and growth. Staining targets were selected from stem cell markers (CD133, CD44, ALDH1), metastasis (PDGRFβ), differentiation (β-Catenin, ERK1/2, p-ERK, HER2, c-MET, Src, p-Src), dormancy (mTOR, P38, p-P38), migration (SMA, E-cadherin, p-JNK), immune resistance (CD45, TGFβ, PD-L1, IL-10, TNF-α, p53, p21), along with Cleaved-Caspase3. Similar to our previous findings, we re-confirmed that despite being a single cell line, there were some rare CSV+ cells within a larger field of population of the same cell line, showing heterogeneity within a cell line. The heterogeneity of protein expression between CSV+ and CSV- cells is illustrated as a panel of selected OHS single cell multiple protein array (Fig. 2a, b). We found that the differences in staining intensity between CSV+ and CSV- OHS was statistically significant for CSV and SMA (Fig. 2b). Our previous data has also shown that these cell states can be transient and will respond to positive or negative CSV state selection to return to the previous equilibrium, indicating presence of self-programming to maintain the same percentage of CSV+ cells within the same cell line. To understand these phenomena, we turned to bioinformatics-based technical analysis to better understand the data. t-SNE-based scatter plots indicated that the cells immediately neighboring the CSV positive TC71 cells harbored a distinct protein signature (Fig. 3a, b). This distinct signature was based on the staining analysis of multiple targets, including, β-Catenin, cleaved caspase 3, PD-L1, p53, PTEN, ERK, CD133, p21, p-p38, Src, p-Src, PDGFRβ, mTOR, m-Myc, IL-10, TGFβ, EpCAM, and TNFα; though the figure panel only shows nuclear and CSV staining for visual clarity. The specific cells whose protein levels are outliers from most other cells show a separate cluster in t-SNE scatter plot (Fig. 3c, d). This indicated CSV+ may influence neighboring tumor cells’ protein expression, causing a distinct and as yet unknown changes. We suspect this interaction may play a role in the disparate states of SMA presence within the CSV+ and CSV- cells as seen in Fig. 2b and c.

### Clinical relevance

While our discoveries regarding the sarcoma cell lines are intriguing, they have limited clinical relevance. To further develop towards our overall goal of precise rare cell detection for prognostic relevance, we used < 500 PDX cell line cells developed from therapy-resistant sarcoma patients; we expected these cells to exhibit greater heterogeneity. Whereas in TC71 cells where the CSV- cells immediately surrounding the CSV+ cells were found to be distinct (Fig. 3a, c), in osteosarcoma PDX cell line M36 (Fig. 3b, d) it was the cells strongly positive for CSV that shows distinct protein staining signature as illustrated by secondary clustering in tSNE distribution pattern (magenta arrows). Both the TC71 and PDX cell lines M36, M31 (data not shown) showed these outlier cells cluster separately as an independent group (Fig. 3c, and d, respectively). However, it is also important to note that not all CSV+ cells were identified as deviant from the mean according to the bioinformatics analysis. This could be a result of lower density of cells used during experimentation, which would minimize cell-cell interactions or the clinical background of the cell lines used. To test our hypothesis, we needed to test a more clinically relevant model than PDX cells.

### Rare cell analysis

**Fig. 4:**
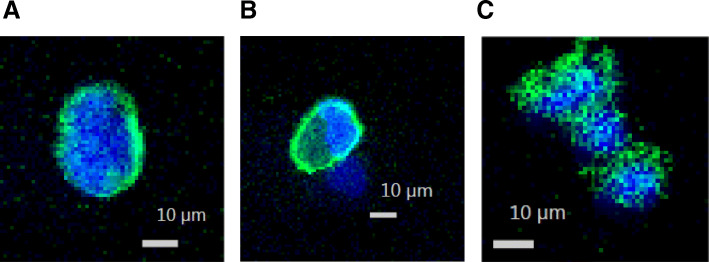
(2 columns): (**a**-**c**) CTC isolated from patient blood. Blue stain is nucleus; Green stain is CSV. Images are representative of multiple panels

**Fig. 5:**
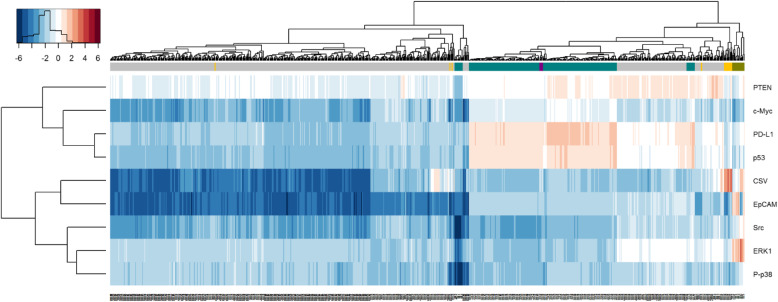
Heatmap of all cells and markers. Gray, teal, olive represents PDX, cell line and CTC samples, respectively. Gold and purple highlight CSV positive cells from the PDX and cell line samples, respectively

**Fig. 6:**
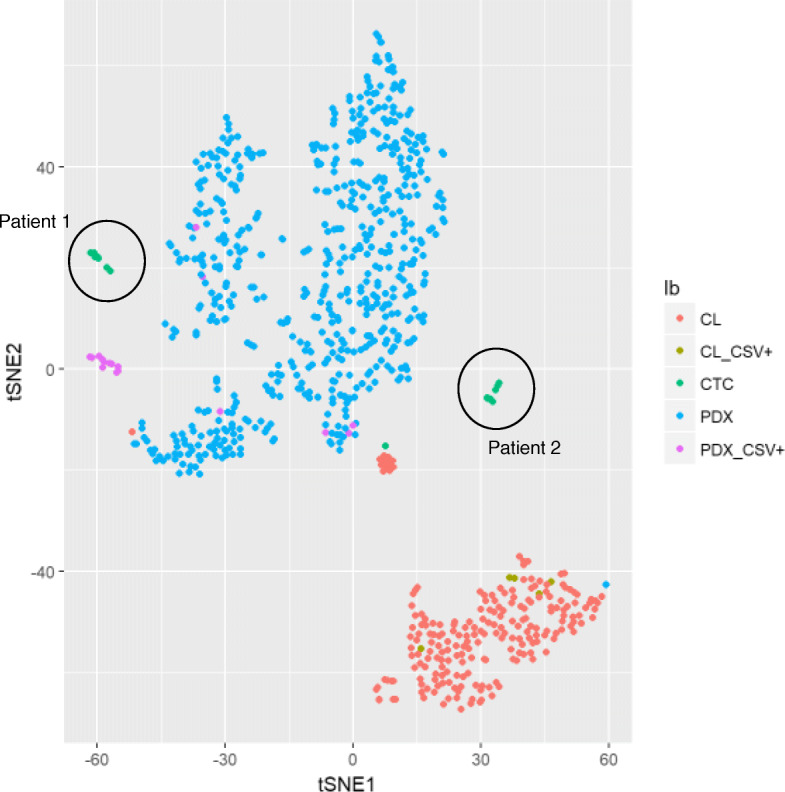
tSNE of all analyzed cells in sarcoma cell lines (CL; red), PDX cells (blue) and CTCs (green)

We analyzed CTCs captured from metastatic sarcoma patients’ peripheral blood. We isolated single CTCs as well as CTC clusters, all of which stained positive for CSV and exhibited variable staining of other markers included in the staining panel (Fig. 4a). Unfortunately, the technological limits of IMC only allows for a resolution limit of 1 μm per pixel [18]. While some subcellular localization may be determined, the image quality is not consistently sufficiently clear enough to do so for single cells, as evidenced in Fig. 4a. We’ve previously noted that sarcoma CTCs detection is highly sensitive to our CSV-based method [4, 27]. Meanwhile, low PTEN in CSV+ CTCs and PDX cells could be an indicator of cell senescence or reduced proliferation as these cells take on a more aggressive phenotype marked by increased invasiveness and migratory potential (Fig. 5). Interestingly, combined suppression of p-53 and PTEN has been previously discovered to induce invasive prostate cancer [28, 29]. Taking a closer look, we asked if the CTCs correlate closely together depending on the patient and if this variable protein pattern will be highlighted by a deeper tSNE-based analysis. As revealed in Fig. 6, there is minimal overlap in overall cell protein signature between the two groups of cells (from two different patients). These outlier circulating tumor cells are often CSV+ which were found to be metastatic in mouse model and highly expressed in metastatic tumor cells from colon tumor patients [30, 31]. Using our approach, it is possible to analyze protein staining data derived from IMC and identify rare cells within a highly limited cell population, as low as 100 or fewer cells.

## Discussion

We employed IMC imaging combined with rare cell-tailored bioinformatics analysis to categorize identify outliers among tumor and PDX cell line populations. We used the gathered protein intensity data to identify outlier cells in a small pool of cells (< 500). We envision our approach can be used to profile or identify rare cells within a low cell number pool such as a FNA. This IMC-based protein profiling could help us learn of active signaling pathways and unique signatures that may indicate future tumor behavior. Ideally, profiling rare cells in FNA would help us deteming the degree to which CTCs recapitulate similar protein signature to the rare cells or the dominant population in a tumor. We found that most tumor analysis methods such as needle biopsies are either invasive or bring high risk to patients for such a study or do not fit our needs [32, 33]. Comparatively, CTCs are highly clinically relevant since they are primary ambassadors of the source tumor, representing more aggressive cells [2, 34, 35]. Therefore, we chose to study CTCs which are easily acquired from peripheral blood and can be quickly studied using immufluorescence or IMC methods.

Our approach can be applied to any rare cell populations that are commonly studied via immunofluorescence methods. Standard techniques rely on either genetic screening or limited-marker based protein profiling [34]. Our method significantly improves the available information that may be extracted from limited, but valuable sources. Besides CTCs, other rare cells such as stem and progenitor cells may also be worthy areas for exploration of protein-based location imaging as we have outlined in our method. Niche subpopulations of immune cells that require large volumes of patient blood for isolation and examination via flow cytometry may also be studied using this protocol. Overall, the cell-analysis application we have presented is relevant for any type of cells that are limited in number and where researchers require the analysis of several targets that would ordinarily necessitate multiple rounds of fluorescence staining and imaging. This new approach may be able to complement, or with further development, even simulate the results of tissue biopsies. Such possibilities would be immensely beneficial for clinical care since liquid biopsies are minimally invasive.

Current fluorescent marker technology is limited to 4–6 targets per stain, depending on the detection equipment. While there are strategies to bleach and re-stain, or other means of antibody stripping from the epitope, these methods are not accepted to be completely clean and effective in comparison to fresh staining [36]. Furthermore, repeated cycles of chemical treatments to re-stain the same cells add the risk of physically changing cell membrane profile. Meanwhile, genetic and flow cytometry-based analysis of individual captured cells cannot ascertain sub-cellular protein localization and co-localization, in addition to being time- and cost-prohibitive [37]. Despite the ability to label several targets, flow cytometry is not relevant for rare cell populations such as CTCs which may only be present in concentrations as low as a single cell per million erythrocytes [2, 38].

While our protocol solves the fast and multiplex analysis of rare cells, it is limited by the constraints of the first generation of IMC machines [18, 23]. The primary limitation is the image resolution, which cannot exceed 1 μm per pixel and significant background noise and non-specific binding. The low imaging resolution prevents a significantly close analysis afforded by confocal systems-based microscopy, while the high background noise makes it difficult to detect true staining. In fact, we observed less than ideal nuclear staining for many of our samples, and often saw artifacts with non-specific binding of the antibodies. The non-specific noise issue may be addressed with better antibody screening, however we found the low nuclear staining problem quite difficult to address. Moreover, the process of scanning/imaging a cell is a “one-shot” execution where the laser will completely burn/ablate the cells while imaging. Therefore, a repeated pass or an adjustment for a better image cannot be made after the first and only pass. Additionally, while the theoretical limit of IMC is > 100 co-labels, the current Fluidigm system advertises a maximum target readout of 37. Another concern of using metal ion conjugates as reporting markers is the small, but noted signal spillover between neighboring molecular weights [39]. To address this concern, signal spillover from each mass used in antibody-metal ion conjugates was recorded by the IMC core facility. The signal spillover readings were incorporated and used for data compensation when analyzing raw protein signal intensity data. We did not observe and significant change in the data or the overall conclusions as a result of these analytical adjustments.

We expect that technological improvements over time will address the speed, background noise issues, and localization accuracy of IMC analysis. Next generation IMC hardware will surely increase the co-label limit and increase scan speed, which is not much of a concern even in its current iteration. We anticipate that with wider adoption and interest, the assay-associated costs will also become lower as well.

## Conclusion

This method helps us make greater inroads towards generating patient-specific CTC fingerprinting that could provide an accurate tumor status from a minimally-invasive liquid biopsy.

## Data Availability

The data that support the findings of this study are available from the corresponding author upon reasonable request.

## Abbreviations

IMC: Imaging mass cytometry
CTCs: Circulating tumor cells
t-SNE: t-Distributed Stochastic Neighbor Embedding
PDX: Patient-derived xenograft
CNV: Copy number variations
FACS: Fluorescence associated cell-sorting
FNA: Fine needle aspirates
CyTOF: Cytometry time-of-flight
CSV: Cell surface vimentin
SMA: Smooth muscle actin
